# Endovascular treatment of iatrogenic superior mesenteric arteriovenous fistula resulting in recurrent abdominal ascites

**DOI:** 10.1259/bjrcr.20200205

**Published:** 2021-05-01

**Authors:** Amy Ho Ching Wong, Warren K W Leung, Wai Kuen Kan

**Affiliations:** 1 Department of Radiology, Pamela Youde Nethersole Eastern Hospital, Chai Wan, Hong Kong

## Abstract

Superior mesenteric arteriovenous fistulas (AVFs) are rare and are usually caused by previous bowel surgery or blunt abdominal trauma. Patients may be asymptomatic, have non-specific symptoms of abdominal pain, nausea and vomiting or present with symptoms of portal hypertension; some patients may present years after initial surgery or trauma. Traditionally, superior mesenteric AVFs are treated by surgical ligation. However, percutaneous endovascular treatment has become increasingly popular in recent years. Different options of endovascular treatment include coil embolisation, covered stent and vascular plugs. There is a risk of coil migration with coil embolisation and covered stents may cause abnormal vessel straightening. Vascular plugs allow the fistula to be treated with fewer devices and have minimal risk of migration. Newer devices such as microvascular plugs have the added advantage of being able to be delivered through microcatheters or diagnostic catheters. The smaller profile of the microvascular plug also allows it to navigate through tortuous vessels. We report a case of a 77-year-old patient presenting with recurrent abdominal ascites three years after small bowel resection. CT and angiogram demonstrated a superior mesenteric AVF, which was successfully treated with a combination of microvascular plug and coil. He remained relatively asymptomatic four months after treatment.

## Clinical presentation

A 77-year-old gentleman presented to gastroenterology outpatient clinic with two-month history of gradual onset of abdominal distension, abdominal pain and loss of appetite. He denied any jaundice, tea coloured urine, pruritus, swelling or weight loss. His past medical history included hepatitis B infection, small bowel resection three years ago secondary to small bowel ischaemia and hypertension. His drug history included spironolactone, entecavir, pantoprazole and neurobion. He was retired and did not smoke or drink. On examination, he had a midline laparotomy scar; his abdomen was soft and not tender but was distended with shifting dullness. He was not jaundiced and did not have hepatosplenomegaly. No abdominal bruit was detected. His blood results including liver function and albumin level were normal. He last underwent an abdominal ultrasound a year ago, which demonstrated liver parenchymal disease with small amount of abdominal ascites. He also had an oesophago-gastro-duodenoscopy earlier this year which demonstrated Grade 1 oesophageal varices and gastritis with erosions. As he was symptomatic from his ascites, he was admitted for abdominal paracentesis and 4L of ascites was drained. His ascitic fluid showed a normal white cell count with negative gram stain and culture. His symptoms improved after the abdominal paracentesis and he was discharged. However, a week later, he presented again with symptomatic ascites. Given the rapid re-accumulation of his ascites, he was admitted for further investigations and a contrast enhanced computed tomography (CT) was arranged.

## Imaging findings

**Figure 1.**
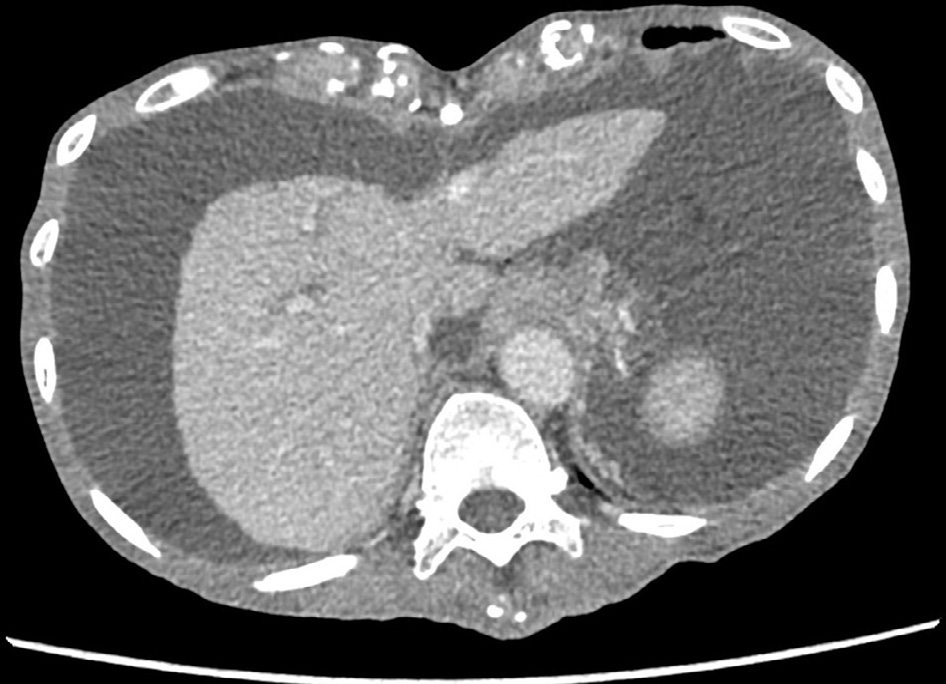
Contrast-enhanced axial CT abdomen shows a small and cirrhotic liver with gross ascites.

**Figure 2.**
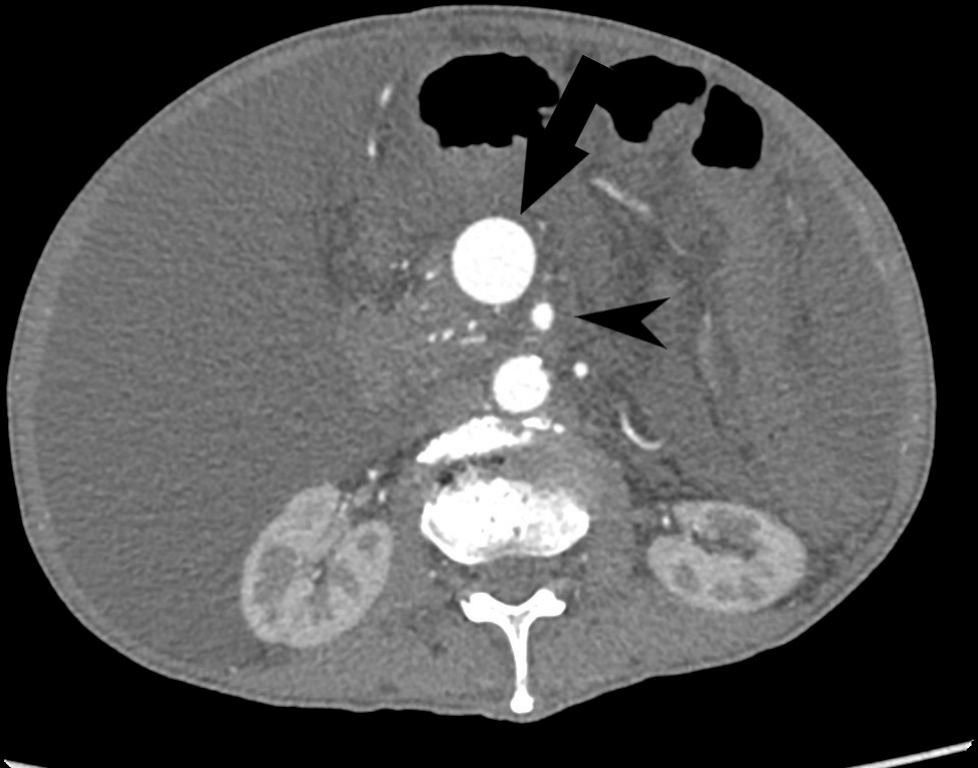
Contrast-enhanced axial CT (arterial phase) shows early enhancement of the superior mesenteric vein (arrow) which is grossly distended and also shows similar enhancement to adjacent superior mesenteric artery (arrowhead).

**Figure 3.**
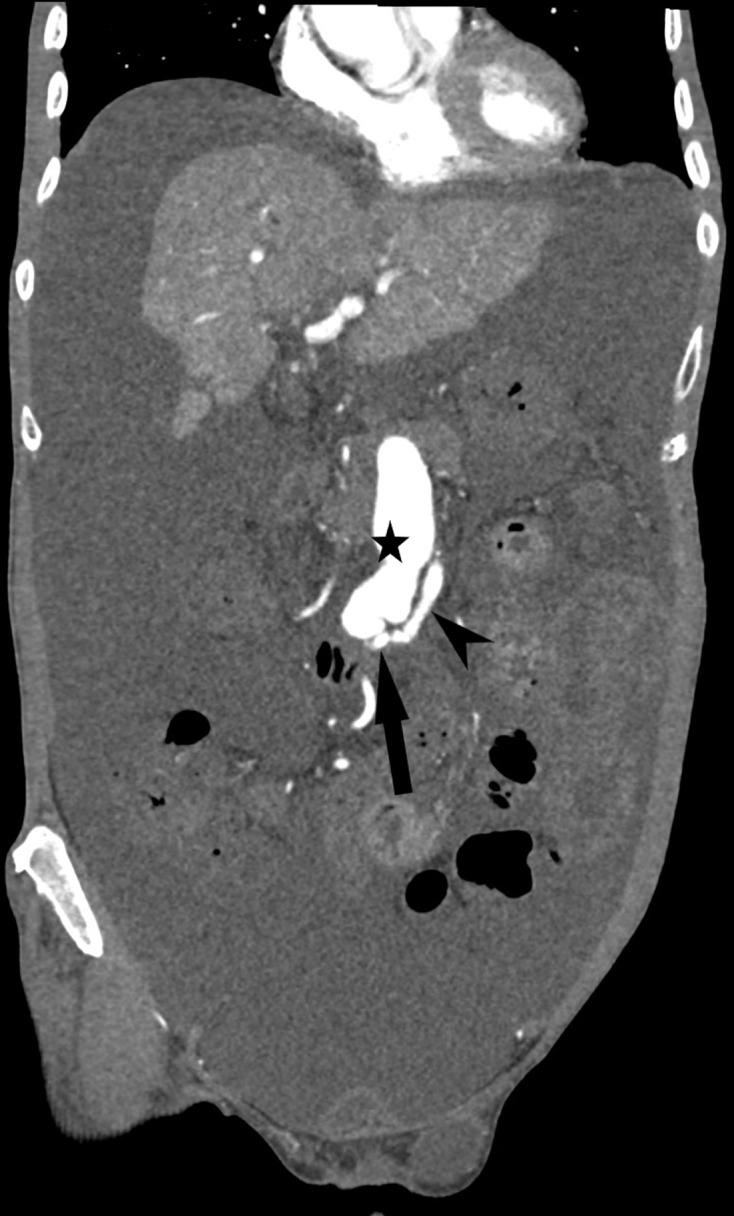
Contrast-enhanced CT with coronal reconstruction (arterial phase) shows a fistula (arrow) between superior mesenteric artery (arrowhead) and grossly distended superior mesenteric vein (star).

Contrast-enhanced CT abdomen showed a cirrhotic liver with gross ascites (Figure 1). In the arterial phase, there was early enhancement of the portal vein and superior mesenteric vein (SMV) which were both grossly distended (Figure 2; SMV is indicated by the arrow; superior mesenteric artery by the arrowhead). There was also an abnormal communication between the middle segment of the superior mesenteric artery (SMA) and the SMV suggestive of arteriovenous fistula (AVF) (Figure 3; the fistula is indicated by the arrow; SMA by the arrowhead and SMV by the star).

## Treatment

**Figure 4.**
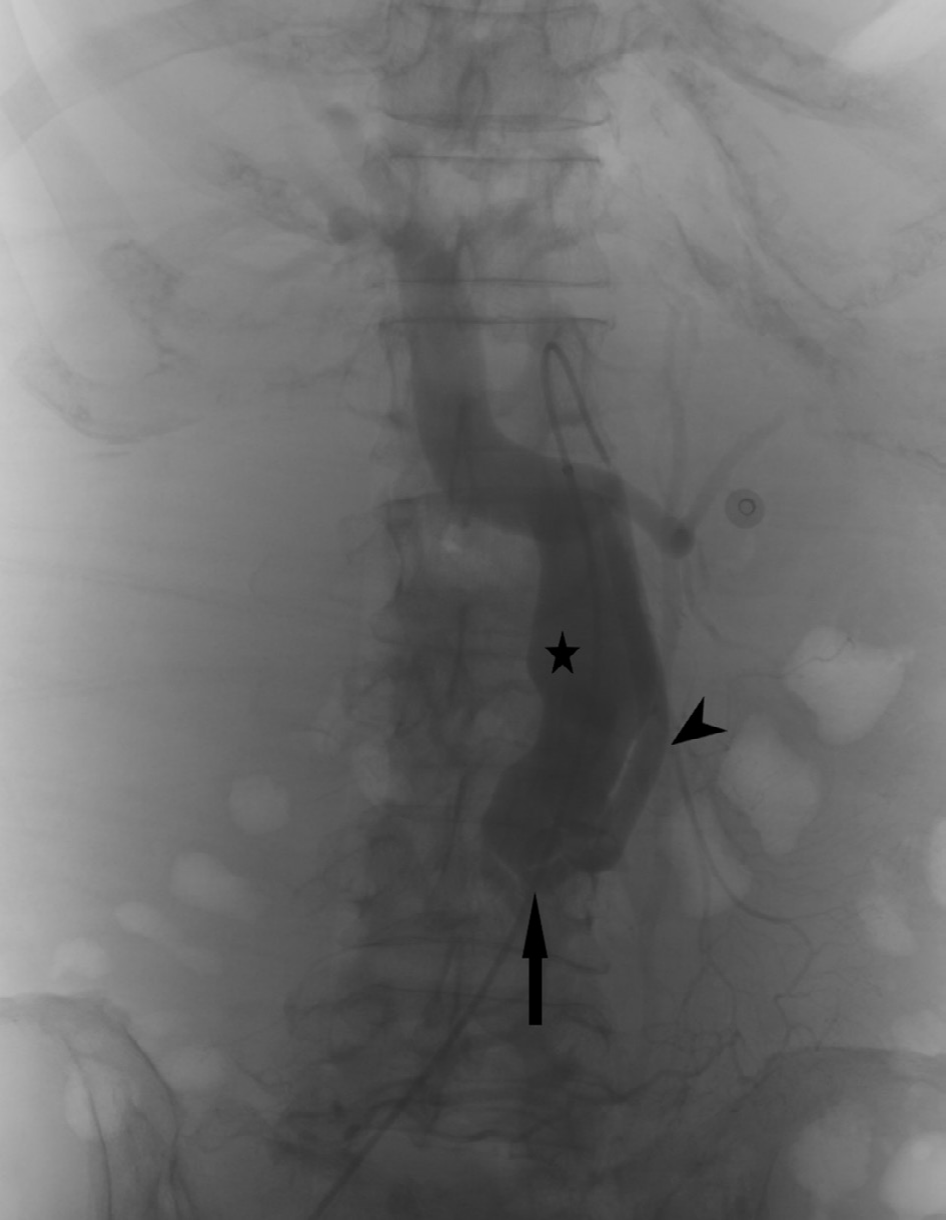
Selective angiogram of superior mesenteric artery shows a fistula (arrow) between the middle segment of superior mesenteric artery (arrowhead) and superior mesenteric vein (star). There is also poor arterial flow to the distal SMA branches.

**Figure 5.**
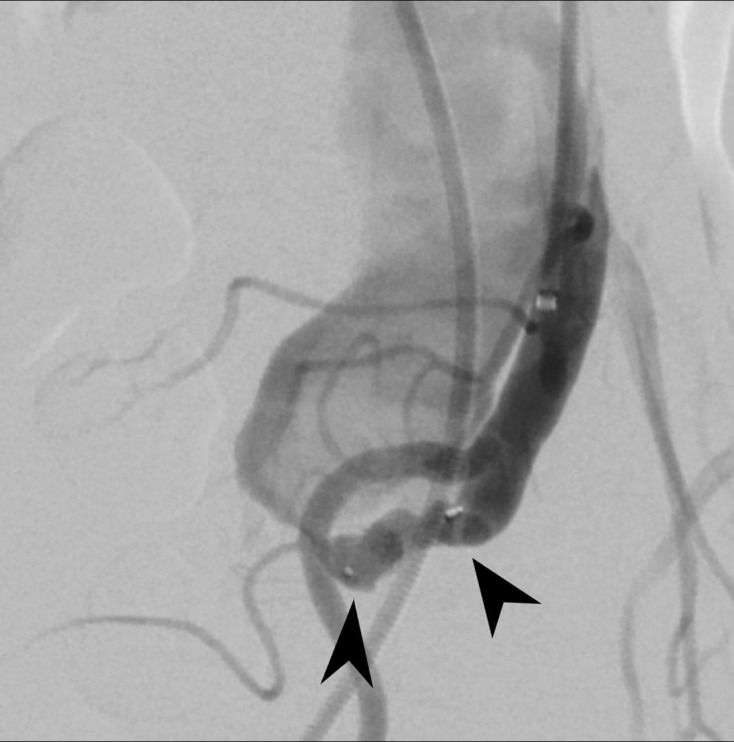
Selective angiogram of superior mesenteric artery at the site of fistula. Microvascular plug (arrowheads) is deployed within the fistula with reduction of blood flow to the superior mesenteric vein.

**Figure 6.**
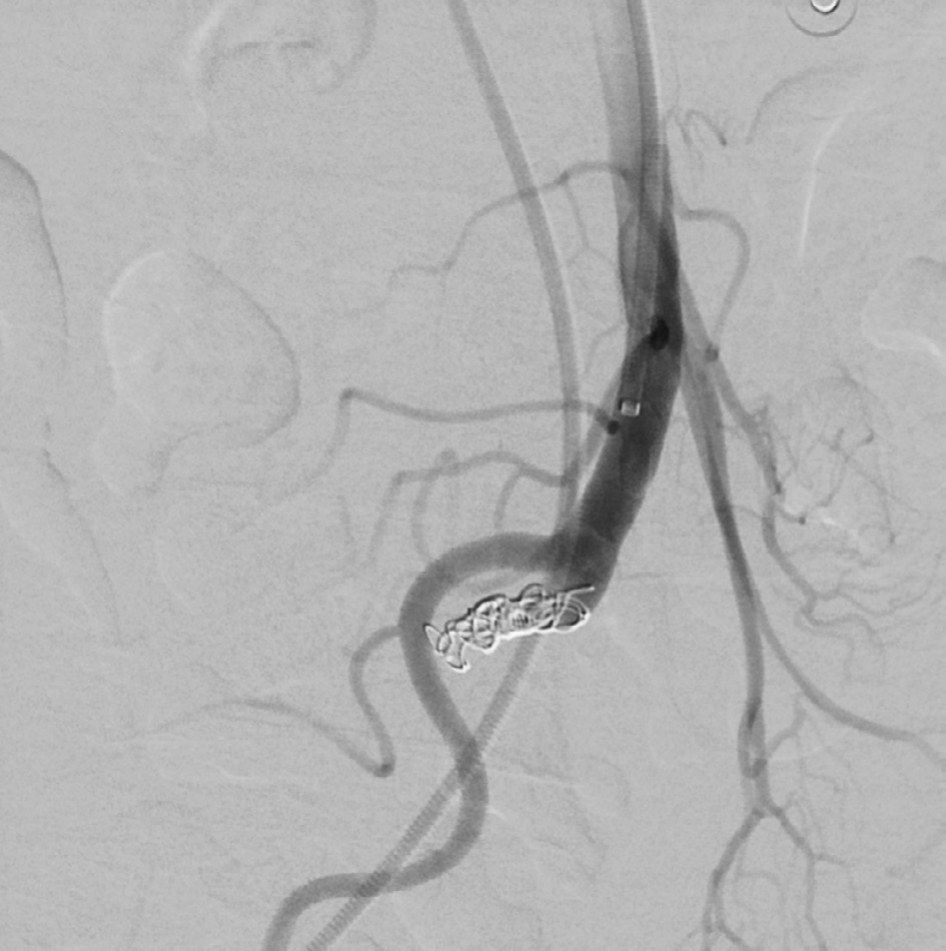
Selective angiogram of superior mesenteric artery at the site of fistula after coil embolisation. There is complete occlusion of the fistula with return of normal blood flow to distal branches of the superior mesenteric artery.

Patient was referred to the interventional radiology department for diagnostic angiogram and endovascular treatment of the superior mesenteric AVF. A 5 French (Fr) 55 cm Flexor^®^ Ansel guiding sheath (Cook Medical, Indiana USA) was first inserted via the right common femoral artery and angiogram of the SMA was performed via a 5 Fr 100 cm Simmons one catheter (Cook Medical, Indiana USA). A fistula between middle segment of the SMA and the SMV was confirmed with poor arterial supply to the distal branches of the SMA (Figure 4; fistula is indicated by arrow; SMA by the arrowhead and SMV by the star). The Flexor^®^ Ansel guiding sheath was then advanced to middle segment of SMA close to the fistula and the fistula was accessed with a 2.7 Fr 130 cm Progreat microcatheter (Terumo, Tokyo Japan). Initial attempts at packing the fistula with a 6mmx20cm Interlock Fibered IDC Occlusion System (Boston Scientific, Massachusetts, USA) was unsuccessful due to the high flow fistula with repeated migration to the SMV; as the embolisation coil of the system was detachable, it was possible to retract the coil before its final replacement. The SMV was then accessed by a 4 Fr H1 catheter (Terumo, Tokyo Japan) and a MVP-7Q microvascular plug (Reverse Medical, California USA) was then deployed within the fistula. The fistula was then further occluded with two detachable Target 360 SOFT 7 mm x 20 cm and 8 mm x 30 cm coils (Stryker Neuorovascular, MichiganUSA) through a 1.7 Fr Excelsior XT-17 microcatheter (Stryker Neurovascular, Michigan USA) (Figure 5; microvascular plug is indicated by two arrowheads). Post-procedure angiogram showed disconnection of the fistula with restoration of arterial supply to the distal SMA branches (Figure 6).

## Outcome and follow-up

Patient was discharged three days after embolisation. At four-month follow-up, his symptoms of abdominal distension and pain have improved and was found to have mild ascites only on examination. His latest blood results also showed normal liver function.

## Discussion

Superior mesenteric arteriovenous fistulas (AVF) are uncommon and most reported cases are associated with previous bowel surgery or abdominal trauma such as gunshot wounds or blunt abdominal injury.^1^ In our patient, the aetiology of the fistula is likely to be due to previous small bowel resection. In bowel resection, it is thought that a transfixation suture passing through the artery and vein simultaneously may result in the formation of the fistula.^2^ Another theory is after continuous ligation of an artery and a vein, a micro-abscess may form, which may result in the breakdown of the vessel walls forming a fistula between the two vessels.^2^

Patients with superior mesenteric AVF often have a delayed presentation which may take place up to fourteen years after bowel resection.^3^ The abnormal communication between the high pressure SMA and low pressure SMV allows blood to bypass the intestinal capillary bed resulting in portal hypertension with patients presenting with ascites or variceal bleeding. Mesenteric steal phenomenon may also occur due to the presence of the fistula.^2,4^ This phenomenon was observed in our patient; on the initial selective arteriogram, there was reduced blood flow to the distal branches of the SMA; after successful occlusion of the fistula, there was return of normal blood flow to these branches. Apart from features of portal hypertension, patients may also be asymptomatic, present with symptoms of abdominal pain, diarrhoea, nausea and vomiting and occasionally, features of right heart failure.^5^

As superior mesenteric AVFs are very rare, a high level of suspicion is required for its diagnosis. It has been suggested that in patients with oesophageal varices and a history of previous bowel surgery, the presence of an abdominal bruit should raise suspicions of this diagnosis.^6^ However, as seen in our patient, abdominal bruits may not always be present. In patients with suspected superior mesenteric AVF, CT is often the first line of investigation; however, it may not always be possible to identify the precise location of the fistula and selective arteriography is required to determine the exact location of the fistula and the extent of mesenteric vessel involvement and also to guide further management.^7^

Superior mesenteric AVFs may be managed by surgery or endovascular treatment. Given the recent advances in interventional radiology, endovascular treatment is often preferred especially in patients who have had previous abdominal surgeries as extensive adhesions may make surgical treatment more challenging.^1,3,8^ The objective of endovascular treatment is to obliterate the fistula without occluding the branches of SMA. One of the considerations when choosing appropriate embolisation method is the type of the fistula: iatrogenic fistulas are usually U type with no distal bowel supply whilst traumatic fistulas are typically H type with distal bowel supply.^2,9^ The options of endovascular treatment include coil embolisation, covered stent and vascular plugs. In coil embolisation, special considerations need to be paid to avoid inadvertent coil deployment and coil migration. Coils may embolise distally into the portal vein resulting in portal vein thrombosis or embolise to the distal SMA or back to the aorta leading to bowel infarction or lower limb ischaemia.^10^ These complications may be avoided by precise measurement of the diameter of the feeding artery and deployment of the coils using a microcatheter system.^9^ An inflated balloon catheter may also be placed proximal to the fistula to prevent coil embolisation.^11^ When covered stents are used, it is important that there is good expansion of the stent and adequate apposition to the vessel wall, in order to minimise the risk of stent thrombosis. If a balloon expandable stent is placed across a sharp bend, it may cause abnormal vessel straightening, vessel injury or stent deformation.^10,12^ To prevent stent thrombosis, patients are put on long term dual antiplatelet therapy. Amplatzer Vascular Plug (AVP) has also been used to occlude superior mesenteric AVF;^5^ unlike coil embolisation, it has minimal risk of migration even in high flow situations or short landing zones; it also allows occlusion of a larger vessel with a single device rather than multiple coils.^13^ However, it does require larger guiding catheters or delivery sheaths. The microvascular plug is a relatively new embolisation device; it allows rapid occlusion, can be re-sheathed and repositioned prior to its release; furthermore, it can be navigated into microcatheters and tortuous vessels. In our patient, initial attempts to occlude the fistula with detachable coils were unsuccessful due to repeated coil migration to the SMV secondary to the high flow fistula. With the aid of the microvascular plug, the blood flow of the fistula was reduced before the deployment of coils, allowing the successful obliteration of the fistula.

## Learning points

- Superior mesenteric AVFs are rare high flow fistulas, which may be managed by surgery or by endovascular treatment.
- It is important to consider the anatomy and configuration of the fistula when choosing the type of endovascular treatment, for example, covered stents may lead to abnormal straightening of vessels in a U type fistula and there is a risk of coil migration with coil embolisation.
- Microvascular plugs are relatively new devices which can reduce the flow of the fistula even in tortuous vessels, allowing the fistula to be occluded by a single device or allow the deployment of coils without the risk of migration.
